# High mobility and high stability glassy metal-oxynitride materials and devices

**DOI:** 10.1038/srep23940

**Published:** 2016-04-05

**Authors:** Eunha Lee, Taeho Kim, Anass Benayad, Jihyun Hur, Gyeong-Su Park, Sanghun Jeon

**Affiliations:** 1 Analytical Engineering Group, Samsung Advanced Institute of Technology, Samsung Electronics Corporation, Suwon 443-803, Republic of Korea; 2 Department of Applied Physics and Department of Display and Semiconductor Physics, Korea University, 2511, Sejongro, Sejong, 446-712, Republic of Korea

## Abstract

In thin film technology, future semiconductor and display products with high performance, high density, large area, and ultra high definition with three-dimensional functionalities require high performance thin film transistors (TFTs) with high stability. Zinc oxynitride, a composite of zinc oxide and zinc nitride, has been conceded as a strong substitute to conventional semiconductor film such as silicon and indium gallium zinc oxide due to high mobility value. However, zinc oxynitride has been suffered from poor reproducibility due to relatively low binding energy of nitrogen with zinc, resulting in the instability of composition and its device performance. Here we performed post argon plasma process on zinc oxynitride film, forming nano-crystalline structure in stable amorphous matrix which hampers the reaction of oxygen with zinc. Therefore, material properties and device performance of zinc oxynitride are greatly enhanced, exhibiting robust compositional stability even exposure to air, uniform phase, high electron mobility, negligible fast transient charging and low noise characteristics. Furthermore, We expect high mobility and high stability zinc oxynitride customized by plasma process to be applicable to a broad range of semiconductor and display devices.

Continued progress in oxide semiconductor materials in thin film electronics leads to enable large−area processing, provide multi−functionality and realize high−density devices^1^,^2^,^3^,^4^,^5^. Electronically active oxide semiconductors exhibit various attractive properties such as high mobility (μ: 3–20 cm^2^/v·s) and high stability even at low temperature process^6^, offering fairly good integration compatibility with conventional technology and providing the integration flexibility with other materials for flexible device applications^7^,^8^,^9^,^10^. Thus, this has generated various devices such as flat panel displays^11^,^12^, remote touch displays^13^,^14^, high−resolution image sensors^15^,^16^, and vertically stackable high density devices^17^,^18^,^19^ to high−voltage devices^20^, in which performance, stability, process compatibility and reproducibility are important properties^21^,^22^,^23^. So far, most works in oxide semiconductor community have focused on the employment of metal cation in zinc−series oxide matrix, forming ternary and quaternary oxide semiconductors^24^,^25^,^26^,^27^,^28^,^29^,^30^. which contain metal cations having the *s*−orbitals overlapping and exhibit relatively high electron Hall mobility (3~20 cm^2^/v· s) even in an amorphous matrix; conversely, the approach to introducing the anion control in zinc oxide has been rarely explored^31^,^32^,^33^. Zinc oxynitride prepared by non-metal anion control in zinc oxide possesses high mobility (~100 cm^2^/v·s) due to low effective mass, which is suitable for future thin film electronic devices. In addition, the substitution of oxygen in zinc oxide with nitrogen buries neutral oxygen vacancies by shifting the valence band edge above the oxygen defect levels, resulting in high illumination−bias stress stability^34^,^35^,^36^,^37^,^38^. However, due to the low binding energy of zinc−nitrogen, the stability, the reliability and the reproducibility of the zinc oxynitride materials and devices have been a remaining issue^36^,^37^,^38^. Especially, high nitrogen−concentration zinc oxynitride which is of our interest particularly due to high−mobility is subject to the composition modification toward stable zinc oxide in the post oxygen process or air ambient, because nitrogen, as compared to oxygen, has a weak binding energy with zinc. This causes the rearrangement of metal cation and non−metal anion in the atomic dimension, which makes difficult to reproduce high−mobility and high−stability zinc oxynitride.

Recently, various post processes after oxide semiconductor thin film deposition have been introduced in order to improve device performance and stability^3^,^6^,^11^,^15^,^39^,^40^,^41^. Post process can be performed on gate insulator, semiconductor channel layer or passivation^3^,^6^,^11^,^15^,^39^,^40^,^41^. Examples of the post process include low temperature thermal annealing^3^,^6^,^11^,^15^,^39^, high pressure annealing,^40^ ultra−violet light exposure and plasma process^41^,^42^ Among these processes, low temperature annealing process has been widely used for display devices due to low temperature thermal budget however it is insufficient to perfectly cure the defects located in the interface as well as the bulk material. Recently, in order to improve annealing effect, high pressure annealing process treated on TFT was reported by several groups and the result showed the enhancement of device performance^40^. For high pressure annealing process, the sample needs to be loaded at room temperature then pressure and temperature set to a process condition. Thus it requires long ramp-up and ramp-down time up to a few hours. On the other hand, ultra-violet assisted photon exposure doesn’t necessarily require additional heating process and thus it is suitable for display device formed on glass substrate however it takes a relatively long process time^41^,^42^. Plasma process includes the combined effect of energetic charge particles impingement and vacuum-ultraviolet photon exposure on the film during plasma processing. It was reported that this process influences on the basic properties of oxide device^43^. In our investigation, plasma process was employed to change the bulk properties of as−prepared zinc oxynitride, solving the composition instability of as−prepared zinc oxynitride. Here, we report materials, chemical and structural characteristics of argon plasma −treated zinc oxynitride via X-ray photoelectron spectroscopy (XPS) and transmission electron microscopy. Also we analyzed various electrical and device characteristics of zinc oxynitride TFT by fast I–V, pulsed I–V, transient–current and low–*f* noise measurement methods. The composition stability of the zinc oxynitride material is examined by the chemical shift of N 1*s* core level peak, thereby allowing us to draw a phenomenological picture of the nitrogen out-diffusion. After long time exposure under air, it turns to zinc oxide. In order to solve the instability of zinc oxynitride film, we performed argon plasma treatment on zinc oxynitride, forming stable nano−crystalline phase embedded in amorphous matrix which retards the reaction of oxygen with zinc oxynitride and enhancing electrical and device characteristics of zinc oxynitride TFT. Therefore, the argon plasma process enables to realize high stable, high performance and low noise zinc oxynitride TFT; this will be an important process towards the realization of next−generation high−performance TFT.

## Results and Discussions

Based on the nitrogen concentration and Hall mobility of zinc oxynitride, there is a strong correlation between nitrogen content and mobility values. A high ratio of nitrogen to the mixing gas (argon, oxygen and nitrogen) during the zinc oxynitride preparation results in the formation of high nitrogen−content and high−mobility zinc oxynitride, which is of interest for advanced technological applications. Thus, among several zinc oxynitride films, the materials analysis was focused on the high nitrogen−content zinc oxynitride film. As presented in Supporting Information 1, the difference in contrast in the bright− and dark−field TEM images indicates the formation of different crystalline zinc oxynitride phases. The HR−TEM image and nano−beam diffraction pattern show the presence of a similar interplane spacing distance for the hexagonal zinc oxide and cubic zinc nitride phases, which impedes to verify the exact ratio of the zinc oxynitride structural composition. To probe the exact electronic structure of zinc oxynitride, we conducted a detailed XPS study based on the zinc 2*p*, oxygen 1*s*, and nitrogen 1*s* core peak analysis, as presented in Supporting Information 2. In comparison, we performed the same XPS study on thermally annealed and argon plasma−treated zinc oxynitride. The oxygen 1*s* and zinc 2*p* 3/2 core peaks are assigned to doubly negatively charged oxygen ions and doubly positively charged zinc ions binding energies in zinc oxide materials^44^. The nitrogen 1*s* core peak is assigned to nitrogen in zinc−nitrogen bonding^45^,^46^. The nitrogen 1*s* asymmetry towards higher binding energy is due to nitrogen in the zinc−oxygen−nitrogen bonding. A similar electronic structure was observed in argon plasma−treated zinc oxynitride. Additional information regarding the chemical state of the zinc oxynitride film was obtained from the XPS Auger spectrum, as presented in Supporting Information 3, which revealed that the film is composed of the zinc nitride, zinc oxynitride, and zinc oxide species.

**Figure 1:**
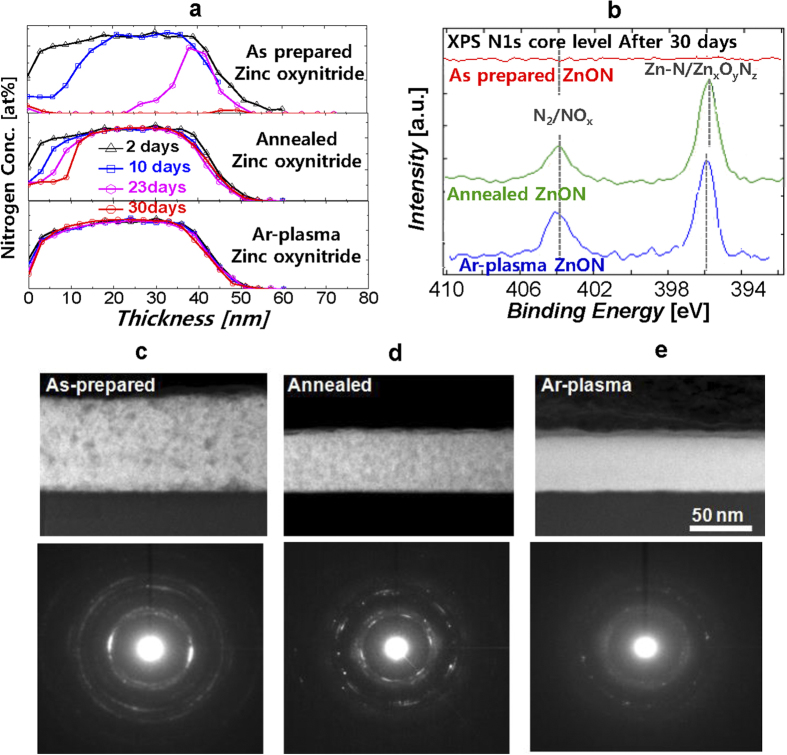
Material characteristics of as−deposited, thermally annealed and argon plasma− treated Zinc oxynitride after exposure to air. (**a**) Depth profile of nitrogen atomic percentage in as−deposited, thermally annealed and argon plasma−treated zinc oxynitride films with respect to air exposure time ranging from 2 to 30 days. After 30−day air exposure, as−deposited zinc oxynitride becomes zinc oxide and the concentration of nitrogen in the film is negligible. (**b**) Depth profile of the nitrogen 1*s* core peak for as−deposited, thermally annealed and argon plasma−treated zinc oxynitride films after one month (30-day) air exposure. Cross-sectional high angle annular dark field scanning transmission electron microscopy (HAADF−STEM) image (top) and diffraction pattern (bottom) of (**c**) as−deposited, (**d**) thermally annealed and e) argon plasma−treated zinc oxynitride films after 30−day air exposure.

Despite the interesting properties of zinc oxynitride, the presence of the relatively weak bonding zinc nitride in the film leads to electronic, chemical and structural modification of as−deposited zinc oxynitride as it is exposed to air, as presented in Fig. 1a. We conducted a systematic XPS spectroscopy depth profile measurement by examining the nitrogen atomic percentage in zinc oxynitride with air exposure time ranging from 2 days to one month. The atomic percentages of other atomic elements, such as oxygen and zinc, are presented in Supporting Information 4. We should note that the amount of nitrogen in as−prepared zinc oxynitride film is gradually decreased with air exposure time and it becomes negligible after one month−air exposure (Fig. 1b). Then, the as−prepared zinc oxynitride becomes zinc oxide, consisting of 1:1 ratio of zinc and oxygen atoms. This indicates that as−deposited zinc oxynitride film after the air exposure induces a significant change in the bulk as well as in the surface. In addition, even after 10 day−air exposure, the zinc−oxygen−nitrogen peak disappears, the zinc−nitrogen peak becomes reduced and nitrogen−nitrogen/nitrogen−oxygen bonding is apparently observed on the surface, suggesting that the nitrogen is migrated toward the surface. The underlying mechanism of nitrogen migration to the surface and the change in the material as well as in the structure can be explained by defect assisted migratio^47^. Also when we take a look at diffraction pattern, the as−prepared zinc oxynitride after 30 day−air exposure forms a wurtzite zinc oxide phase and the color of film changes from dark brownish to see-through, indicating that structure and the optical properties are entirely changed^48^.

For making highly robust zinc oxynitride film, the change in electrical, chemical and structural bonding of zinc oxynitride after air exposure should be minimized. To this end, we employed two different processes such as thermal annealing and argon plasma processes. Then, in order to probe the change in the chemical bonding configuration, we monitored nitrogen concentration−depth profile by XPS measurements for both post processed samples with air exposure time. As presented in Fig. 1a, the slight change of nitrogen concentration was observed for the thermally annealed zinc oxynitride, while there is negligible change of nitrogen concentration with air exposure time for argon plasma−treated zinc oxynitride. Accordingly, even after one month−air exposure, the chemical state of the nitrogen 1*s* core levels for zinc nitride and zinc oxynitride for both the post processed samples remains stable unlike the as−prepared zinc oxynitride as presented in Fig. 1b. Thus, the post process is essential to prepare for highly robust zinc oxynitride film. In addition, the tint of the post processed zinc oxynitride films keeps on transparent brownish.

**Figure 2:**
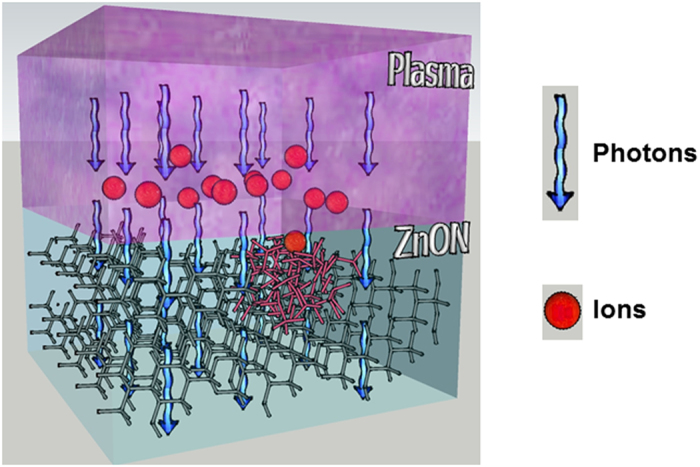
Model of film densification induced by physical ion bombardment and high energy photon exposure during plasma process. Red ball and blue arrow indicate positively charged argon ion and photon.

As displayed in the TEM images and the diffraction patterns (Fig. 1c–e), the annealed zinc oxynitride film shows a different contrast in the HAADF-STEM image and characteristics lines with tiny bright dots in the diffraction pattern, suggesting that the annealed zinc oxynitride film exhibits a quite different chemical bonding configuration and a mixed phase in an amorphous matrix. On the contrary, for the TEM image of the argon plasma−processed zinc oxynitride, there is no contrast change and it only appears haziness in the diffraction pattern, implying that the spatial distribution of crystal structure as well as the chemical bonding configuration of argon plasma−treated zinc oxynitride is homogeneous across the film and this process promotes the formation of amorphous structure. During plasma process, the energetic ions from the plasma inside collide with the film surface and physically bombard the backbone bonds^43^. The combined effect of ion impingement and high energy photon during plasma process changes the properties of bulk film as seen in Fig. 2. According to the spectrum of argon plasma measured with a calibrated monochromator, the energy in UV region was observed to be 5.6–6.2 eV. High nitrogen−concentration zinc oxynitride which is of our interest due to high mobility presents low optical band-gap of 1.3 eV. Thus, when UV photon is exposed to tested zinc oxynitride films of 30–50 nm thickness, the whole film absorbs UV photon.

**Figure 3:**
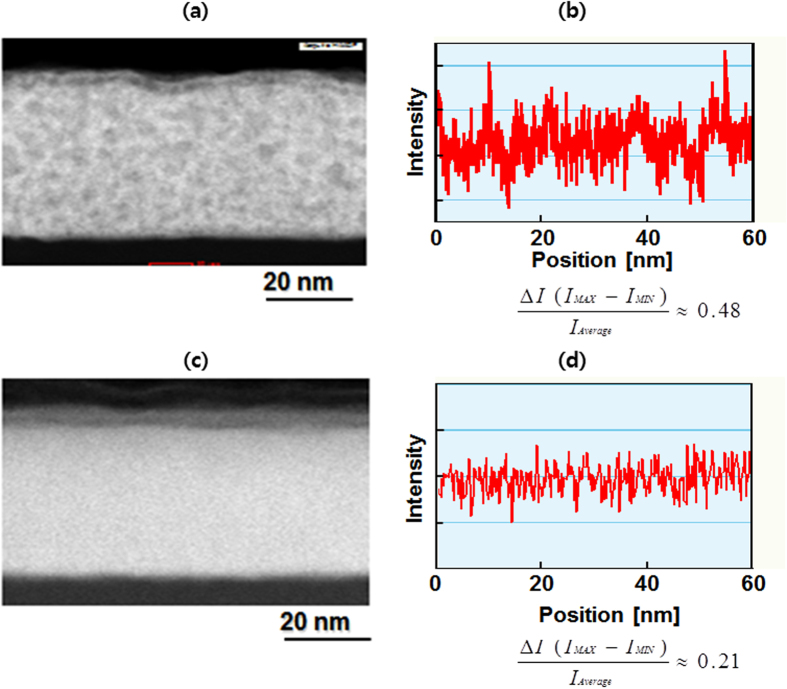
Quantitative analysis on uniformity of thermal annealed and argon plasma**−** treated zinc oxynitride. (**a**) Cross−sectional HAADF−STEM image and (**b**) intensity deviation plot of thermally annealed zinc oxynitride. (**c**) Cross−sectional HAADF−STEM image and (**d**) intensity deviation plot of argon plasma−treated Zinc oxynitride.

To quantitatively measure the spatial uniformity of the bonding structure of post processed zinc oxynitride, we evaluated the intensity (proportional to atomic number) with the position in the high angular annular dark field image in scanning transmission electron microscopy for both the annealed sample and the argon plasma−treated sample (see Fig. 3). The ratios of the intensity difference (ΔI = I_MAX_ − I_MIN_) to the average intensity for thermally annealed zinc oxynitride and argon plasma−treated zinc oxynitride are 0.48 and 0.21, respectively. Argon plasma is reported to foster the collision cascade of both atoms and ions, contributing to rearrangements toward a homogeneous configuration^49^. This report demonstrated the growth by atomic redistribution with the formation of a homogeneous alloy. In our work, the argon plasma process causes the densification resulted from atomic and ionic rearrangement processes, thereby forming nano−crystalline phase embedded in glassy matrix, as verified by the STEM−HAADF image. We also evaluated electrical properties of various zinc oxynitride films such as as−deposited, thermally annealed and plasma−treated samples with air exposure time by probing the sheet resistance (R_sheet_) of films. Consistent with the evolution of nitrogen 1 s spectrum of zinc oxynitride film with air exposure as presented in Fig. 1, the R_sheet_ of as−deposited zinc oxynitride is dramatically increased with air exposure time, suggesting that the electrical property of as− deposited zinc oxynitride is drastically changed under air ambient. While, the R_sheet_ of thermally annealed zinc oxynitride film with air exposure time is gradually increased, implying that thermal annealing process is not perfect to ensure the stability of zinc oxynitride. On the other hand, argon plasma−processed zinc oxynitride film shows an insignificant change of R_sheet_ of film even with air exposure time, indicating that argon plasma−treatment process is the most appropriate post process to form highly robust film. As expected, we found that the as−deposited zinc oxynitride device was not working due to instability and thus we measured device characteristics of only post−processed zinc oxynitride TFTs.

**Figure 4:**
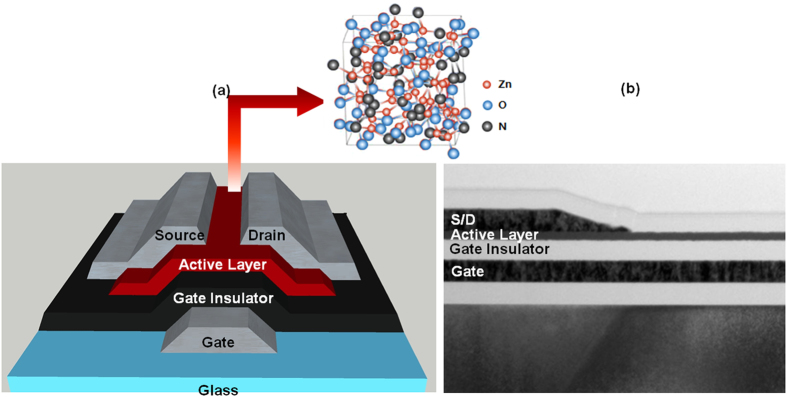
(**a**) Schematics and (**b**) transmission electron microscopy image of zinc oxynitride thin film transistor.

For future semiconductor and display devices with high performance and high density, the carrier mobility in semiconductor should possess 100 cm^2^/v·s and above^34^,^35^. We fabricated zinc oxynitride TFTs as presented in Fig. 4. To characterize the channel mobility of zinc oxynitride TFTs, the transfer characteristics (I_DS_–V_GS_) of TFTs were measured at the drain bias of 10 V. Conventionally, the channel mobility of the TFTs is extracted from the direct-current I–V measurement data. Since direct-current I–V measurements method takes a certain time such as 1 to 10 seconds or longer, one can’t get rid of the charge trapping issue because of comparatively long test time^50^. When the device turns on, channel carriers can be injected and trapped into interface due to a given gate to channel electric field, reducing the drain current level due to the positive shift of threshold voltage. As a consequence, the mobility values by direct-current I–V test method tend to be underestimated. In order to measure device characteristics under charge–trap free environment, we employed fast and short pulse I–V test (rise time of 1 μs) methods. For comparison, the device performances of the TFTs were measured using direct-current I–V measurement as well.

**Figure 5:**
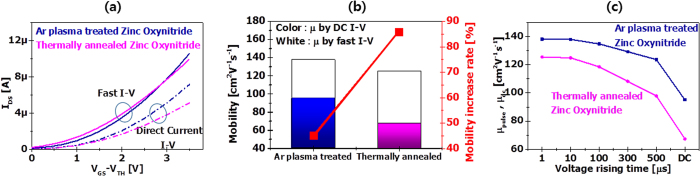
Transistor characteristics of thermally annealed and argon plasma−treated zinc oxynitride TFT. (**a**) Fast I–V and direct-current I–V characteristics of thermally annealed and argon plasma–treated zinc oxynitride devices. (**b**) Mobility values of thermally annealed and argon plasma–treated zinc oxynitride devices obtained by fast I–V and direct-current I–V measurement methods. (**c**) Mobility values of thermally annealed and argon plasma–treated zinc oxynitride devices obtained by fast I–V with respect to voltage rising time and direct-current I–V measurement methods.

Figure 5a displays direct-current and fast I_DS_–V_GS_ curve (rise time of 1 μs) of post-processed TFTs. For both thermally annealed and argon plasma–treated zinc oxynitride TFTs, the fast I–V curve presents higher drain current level and steeper slope than direct-current I–V curve. The drain current levels of argon plasma–treated zinc oxynitride TFT by both direct-current and fast I–V test methods are higher than those of thermally annealed zinc oxynitride TFT. Figure 5b presents the field effect mobility values (μ_FE_) of both thermally annealed and argon plasma−treated zinc oxynitride TFTs extracted from direct-current and fast I–V test methods. We found that the μ_FE fast_ obtained by fast I_DS_−V_GS_ curve is higher than the μ_FE DC_ by direct-current I_DS_−V_GS_ data, implying that fast charging effect can be minimized by the fast I–V measurement, especially ultra-fast (~1 μs) measurement as presented in Fig. 5c 46. If the bias ramping time is shorter than charge trapping time constant, we can obtain the intrinsic I–V characteristics of the zinc oxynitride devices during the rise time. In addition, thermally annealed zinc oxynitride TFT exhibits a relatively higher ratio of mobility (μ_FE fast_/μ_FE DC_) than argon plasma−treated device, indicating that thermally annealed zinc oxynitride TFT is vulnerable to charge trapping and seems to have a relatively high number of interfacial defects or acceptor like defects in active semiconductor (consistent with the compositional non-uniformity observed in the materials data) compared with argon plasma−treated device. This causes the decrease in the μ_FE_ values. The μ_FE_ values of argon plasma−treated and thermally−annealed zinc oxynitride TFT extracted by the fast I–V measurement are 138 and 124 cm^2^/V·s, respectively, whereas those from the direct-current I–V curves of argon plasma−treated and thermally annealed zinc oxynitride devices are 95 and 67 cm^2^/V·s, respectively. The μ_FE_ of argon plasma−treated and thermally annealed zinc oxynitride devices increased by 45 and 85% after the fast I–V measurement, respectively. As presented in Fig. 5c, the mobility values of both devices are gradually decreased with voltage ramping time. This result indicates that the μ_FE_ by conventional direct-current I–V measurement has been significantly underestimated and the fast I–V method should be used to evaluate device characteristics of TFT in order to evaluate the geniune mobility and exclude the effect of the fast charge trapping^52^,^53^.

**Figure 6:**
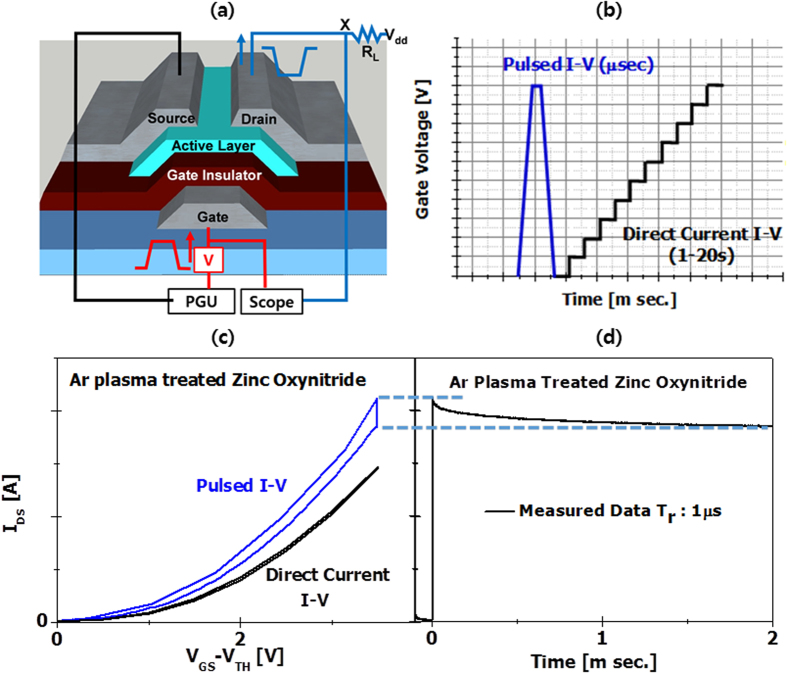
(**a**) Schematics of pulse I–V measurement system (**b**) Voltage ramping profile of direct-current and pulsed I–V methods (**c**) direct-current & pulsed I–V curves and (**d**) transient current characteristics of argon plasma–treated zinc oxynitride TFT.

In order to characterize hysteresis phenomena, short pulse I–V measurements (rise time/fall time of 1 μs and pulse width of 2 ms) were performed as compared with direct-current I–V method, as presented in Fig. 6a and voltage ramping profiles are displayed in Fig. 6b. During forward gate voltage sweep (V_GS:_ 0 V to high), we measure I–V data. During pulse width of 2 ms, the drain current is gradually reduced due to fast charge trapping. Then, during reverse gate voltage sweep (V_GS_ high to 0 V), we measure another I–V curve, as presented in Fig. 6c. Hysteresis characteristics were clearly observed mostly because of the electron charging during the pulse width of 2 ms. The threshold voltage shift (ΔV_th_) values of two I–V data for argon plasma−treated and thermally annealed zinc oxynitride TFT exhibit 0.23 and 0.6 V, respectively. In addition, due to relatively long measurement time during direct-current measurement, the current skew (ΔI_DS_) between direct-current and pulsed I–V test methods is observed as presented in Fig. 6c. Also during pulsed I–V test, transient current characteristic were measured as seen in Fig. 6d.

**Figure 7:**
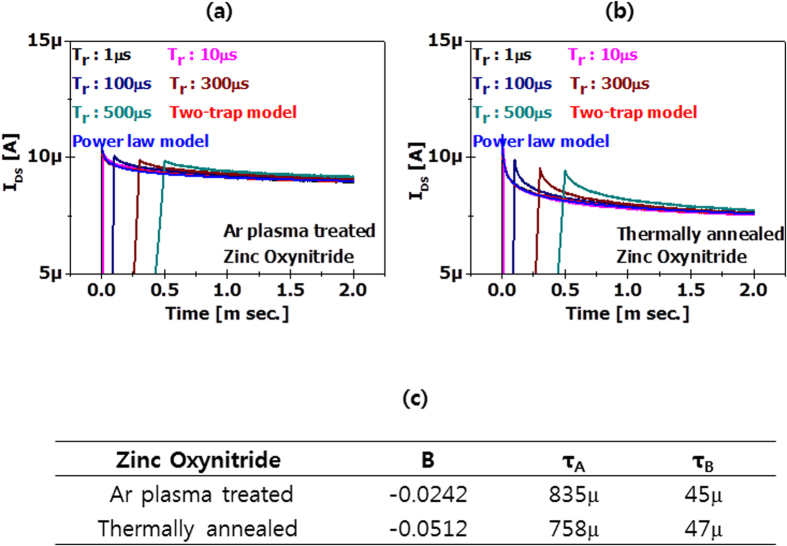
Transient current characteristics of (**a**) argon plasma–treated and (**b**) thermally annealed zinc oxynitride TFTs as a function of voltage rising time. (**c**) Exponent and time constants of argon plasma–treated and thermally annealed zinc oxynitride TFT obtained from power law and two trap models, respectively.

Fast transient drain current data was measured at different rise times ranging from 1 to 500 μs. Figure 7a,b show the reduction in the time−dependent current of argon plasma−treated and thermally annealed zinc oxynitride TFTs a a function of voltage rising time. Immediately after the voltage ramping is elapsed and it reaches a certain gate voltage, the drain current reduction is very rapid at the beginning because of initial fast charge trapping, then it gradually reaches a saturated current level. The measured current data was modeled with a two−trap model, consisting of an exponential function, I = A·I_o_·exp(−t/τ_A_) + B·I_o_·exp(−t/τ_B_) with trapping time constants (τ_A_, τ_B_). Also, it can be modeled with power law using I = A·I_o_·t^B^. The fitting curves exhibit how well these models can match the measured data to each model. Although the power law can’t model the initial trapping data, the two−trap model can explain the early trapping behavior as well as the longer time scale. As the two−trap model appears to fit well both regions, the charge trapping is apparently controlled by multiple processes with at least two different time constants. For argon plasma−treated zinc oxynitride TFT, two time constants, τ_A_ and τ_B_ are 835 and 45 μs, respectively. For thermally annealed zinc oxynitride TFT, τ_A_ and τ_B_ are 758 and 47 μs, respectively.

**Figure 8:**
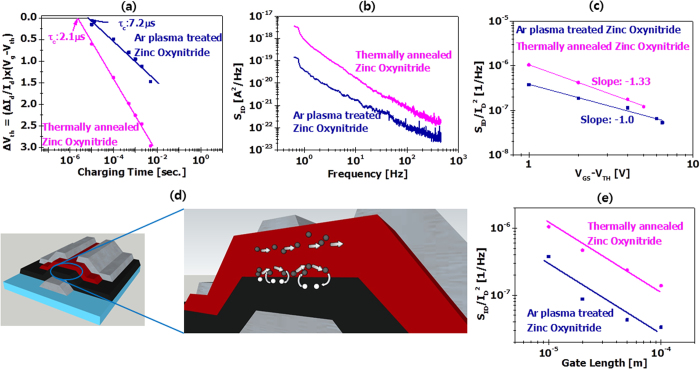
(**a**) Threshold voltage versus charging time plot, (**b**) low frequency noise characteristics, (**c**) normalized power spectral density versus gate drive voltage (**d**) mobility fluctuation (Δμ) and carrier number fluctuation (Δn) models and (**e**) normalized power spectral density versus gate length of argon plasma–treated and thermally annealed zinc oxynitride TFT.

The threshold voltage shift, ΔV_th_, was extracted from the drain current using the equation, ΔV_th_ = ΔI_d_·(V_g_ − V_th_)/I_d_, where ΔI_d_ is the drain current skew between initial and final points of the gate pulse width, I_d_ is the maximum drain current prior to charging, V_g_ is the gate bias amplitude and V_th_ is the threshold voltage. From this equation, ΔV_th_ was determined and is plotted with respect to charging time for both post−processed zinc oxynitride devices. The critical charging time values for argon plasma−treated and thermally annealed zinc oxynitride TFT exhibit 2.1 and 7.2 μs, respectively, as presented in Fig. 8a. At a certain charging time, the charge trapping of thermally annealed zinc oxynitride TFTis more significant than argon plasma−treated device.

To evaluate the conduction mechanism of zinc oxynitride TFT, we conducted low frequency noise measurements for both zinc oxynitride TFTs using a B1500 parameter measurement system with waveform generator and fast measurement unit. One should note that the low frequency noise follow the 1/f^r^ behavior in both devices and lower power spectral density is lower trap density as seen in Fig. 8b. The influence of normalized power spectral density (S_ID_/I_D_^2^) on the net gate voltage |V_GS_ − V_TH_| was characterized^54^ Fig. 8c presents the S_ID_/I_D_^2^ versus |V_GS_ − V_TH_| at a frequency of 10 Hz. For the argon plasma−treated and thermally annealed zinc oxynitride devices, the slopes are −1.0 and −1.3, respectively. A gradient of −2 shows denotes that the carrier-number-fluctuation (Δn) in the interface between gate insulator and semiconductor channel region is responsible for the low-frequency-noise, while a gradient of −1 specifies that mobility fluctuation (Δμ) is responsible for the origin of low frequency noise^55^. Thus, we presume that for Ar plasma−treated zinc oxynitride, the low frequency noises follow the Δμ theory, but for thermally annealed zinc oxynitride, the low frequency noises follow the theory of Δn of correlated Δμ as seen in Fig. 8d. According to the theory of Δn with correlated Δμ^54^,^55^,^56^, the S_ID_/I_D_^2^are related to the number of defects; argon plasma−treated zinc oxynitride is two times lower defect densities than the thermally annealed zinc oxynitride devices. We also investigated the dependence of the gate length on the low frequency noises of the zinc oxynitride devices as seen in Fig. 8e. The same tendency was also observed in all the tested devices with various channel lengths of 10 to 200 μm. The gate length effect on the low-frequency noises is used to determine the noise mechanism, channel noise or contact noise. When the former (channel) noise is dominant, the S_ID_/I_D_^2^ is inversely proportional to channel length. Whereas the contact is responsible for the noise, the S_ID_/I_D_^2^ is inversely proportional to channel length square^51^. The measured gradient of 1.01 implies that the low frequency noise is mainly responsible for the channel region.

Lastly, in order to ensure high stability especially even under the combined stability test condition of light illumination and bias stress, we measured ΔV_th_ of TFT with bias illumination stress time and light intensity. Since as-prepared zinc oxynitride device is conductive, we compared the stability of various processed zinc oxynitride compared to conventional InZnO TFT. Consistent with chemical, electrical and structural characteristics, the stability of Ar plasma treated zinc oxynitride device is found to superior to others as presented in the Supporting Information 5.

## Conclusions

In our investigation, we solved inherent instability issue of zinc oxynitride semiconductor film by performing Ar plasma process, which leads to form nano−crystalline phase embedded in glassy matrix and retards the reaction of oxygen with zinc, as verified by XPS and transmission electron microscopy analyses. Due to stable phase formation, electrical and device characteristics of zinc oxynitride materials/devices were significantly improved, presenting high mobility and negligible charge trapping. From the transient I–V characteristics, the carrier transport phenomena of the zinc oxynitride devices follow a two-trap model, in which the charge trapping is governed by multiple processes with at least two different time constants. In addition, we probed the conduction mechanism of zinc oxynitride by low frequency noise methods. These results demonstrate how the material and process attributes of zinc oxynitride can be optimized for new device strategies in both semiconductor and display technologies. We expect argon plasma treated zinc oxynitride to be applicable to a wide range of high performance, high density, large area and ultra-high definition display devices and systems. In addition, as long as electronically active oxide semiconductors exhibit high mobility exceeding 100 cm^2^/v·s, high stability and low temperature process capability, the application of this material can be further extended to system LSI and memory products, forming vertically stacked three dimensional device.

## Methods

### Materials Preparation

Zinc oxynitride thin films were prepared by reactive radio-frequency (RF) magnetron sputtering system at room temperature using a mixture of nitrogen, oxygen and argon gas as working gases. A zinc metal target was used. In order to adjust the anion content in the films, the oxygen partial pressure was controlled during the sputtering process by varying the oxygen flow rate and the chamber gas pressure (0.4–0.8 Pa), while the radio frequency power (300 Watt), the nitrogen (100 sccm) and argon flow rates (10 sccm) were kept constant. The film thickness was set to be approximately 50 nm.

### Materials Analysis

The chemical structure of the zinc oxynitride films was characterized by XPS method using a focused monochromatic Al Ka radiation. The residual pressure inside chamber was 1.1 × 10^−9^ Torr. The spectrometer with an energy resolution of 0.2 eV was calibrated using gold (Au 4 *f*_7/2_ = 84.0 eV), with Fermi level. The core−level and the valence band spectra were recorded with a constant pass energy of 29.3 eV. The depth profile analysis on the electronic structure of zinc oxynitride was examined by a sequenced argon ion sputtering from the film surface to the bulk inside with 1 keV beam energy with a certain time duration. For comparison, argon gas cluster ion beam sputtering analysis was also performed to overcome the preferential sputtering usually observed in the case of positively charged argon ions sputtering processes for 1 min. In this case, the surface of the samples was bombarded with argon 2500+ clusters accelerated at the potential of 20 kV. To analyze the crystalline phase of the zinc oxynitride thin film, high resolution transmission electron microscopy and nano-beam diffraction pattern analyses were performed. The high angle annular dark field (HAADF) in scanning transmission electron microscopy (STEM) mode was used to determine the compositional distribution of the nanostructure.

### Device Fabrication and Characterization

Zinc oxynitride TFTs were fabricated with bottom gate structure using molybdenum electrodes. 50 nm−thick zinc oxynitride layer was prepared using a sputtering method. A bi−layer, silicon nitride–silicon oxide, gate insulator was deposited on the gate electrode. To evaluate intrinsic device characteristics under charging trap-free environment, fast I–V method was used. In addition, to study the impact of fast charge trapping on the performance of the zinc oxynitride TFT, the short-pulsed I–V measurement was used. Also, low frequency noise measurements were performed using a low-noise amplifier and a dynamic signal analyzer. The details regarding noise measurement method can be found in elsewhere^26^. For the bias-illumination stress measurement, we used a gate bias of 20 V and a drain bias of 10 V to each TFT device at room temperature. A white-light LED with a light intensity was used in order to supply visible light.

## Additional Information

**How to cite this article**: Lee, E. *et al*. High mobility and high stability glassy metal-oxynitride materials and devices. *Sci. Rep.* **6**, 23940; doi: 10.1038/srep23940 (2016).

## Supplementary Material

Supplementary Information
