# Supplementary material for: High mobility and high stability glassy metal-oxynitride materials and devices
Source: Sci Rep. 2016 Apr 5;6:23940. doi: 10.1038/srep23940 (PMC4820723; doi:10.1038/srep23940)
Supplement: Supplementary Information [file srep23940-s1.doc]

## **Supporting Information**

**High mobility and high stability glassy metal−oxynitride materials and devices**

Eunha Lee^1*^, Taeho Kim^2*^, Anass Benayad^1^, Jihyun Hur^2^, Gyeong-Su Park^1^, and Sanghun Jeon^2+^,

1 *Analytical Engineering Group, Samsung Advanced Institute of Technology, Samsung Electronics Corporation, Suwon 443-803, Republic of Korea*

2 *Department of Applied Physics and Department of Display and Semiconductor Physics, Korea*

*University, 2511, Sejongro, Sejong, 446-712, Republic of Korea*

**S1. Transmission Electron Microscope images of zinc oxynitride**

**S2. X-ray photoelectron spectroscopy data of O1*s*, N1*s* and Zn2_p3/2_ core peaks.**

**S3. XPS Zn L_3_M_45_M_45_ Auger spectrum of zinc oxynitride film.**

**S4. Depth profile atomic percentage of nitrogen, oxygen and zinc from XPS quantitative analysis performed after the 1^st^, 8^th^, 15^th^, 23^rd^ and 29^th^ day**

**S5. Reliability characteristics of various TFTs**

* These authors are equally contributed to this work

[+corresponding authors: jeonsh@korea.ac.kr,](mailto:+corresponding authors:  jeonsh@korea.ac.kr,  )

**S1. Transmission Electron Microscopy (TEM) Images of Zinc Oxynitride**


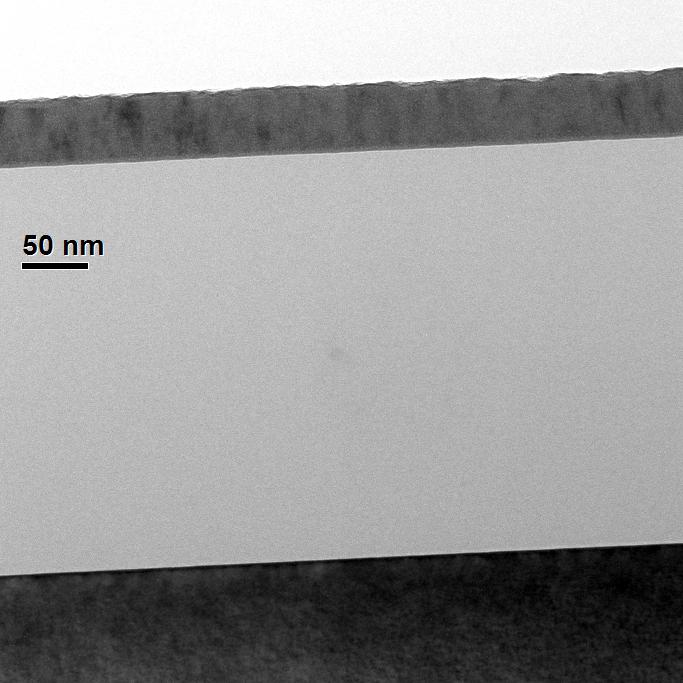


**a)**


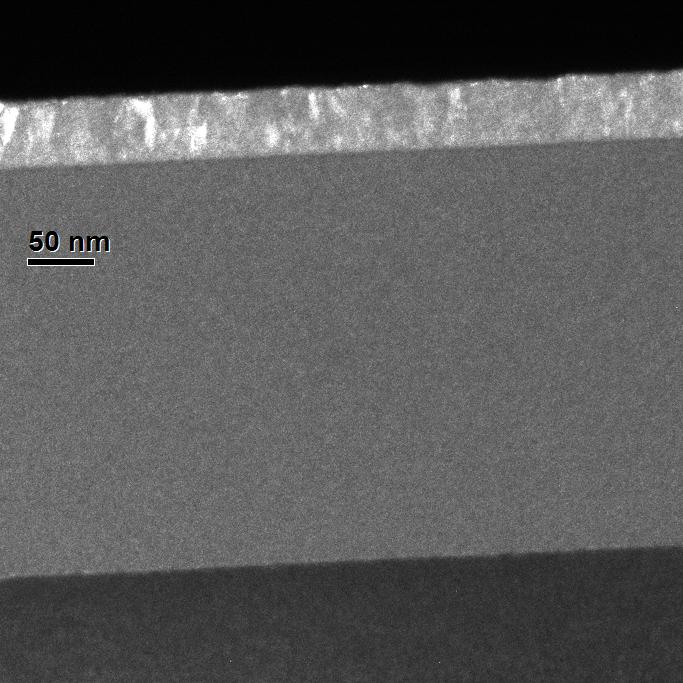


**b)**

**ZnON**

**ZnON**

**SiO_2_**

**SiO_2_**

1. Bright field and (b) dark field TEM images of 50nm-thick zinc oxynitride thin film deposited on SiO_2_/Si substrate.


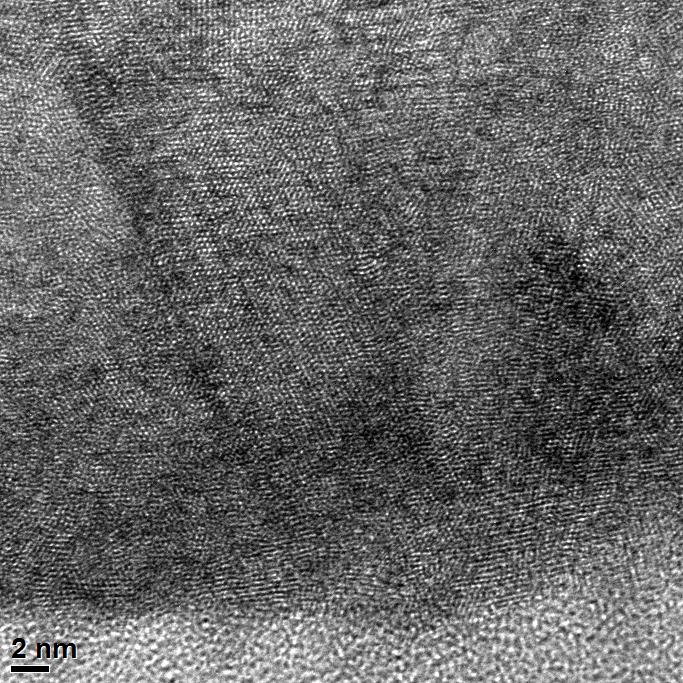


**ZnON**

**SiO_2_**

**c)**

**d)**

(c) High resolution transmission electron microscope image and (d) nano-beam diffraction pattern of zinc oxynitride thin film on SiO_2_/Si substrate [Ref. E. Lee et al., NPG Scientific Reports 4:4948 2014].

**S2. X-ray photoelectron spectroscopy data of O1*s*, N1*s* and Zn2_p3/2_ core peaks.**

(a-b) O1*s*, N1*s* and Zn 2*p* 3/2 core level peaks after 10min Ar ion beam and Ar GCIB sputtering, respectively. The inset figure presents different N1s peak after sputtering with Ar^+^ ion beam energy of 0.5, 1 and 2keV, respectively

**S3. XPS Zn L_3_M_45_M_45_ Auger spectrum of Zinc Oxynitride film. The Wagner plot is presented in the inset.**


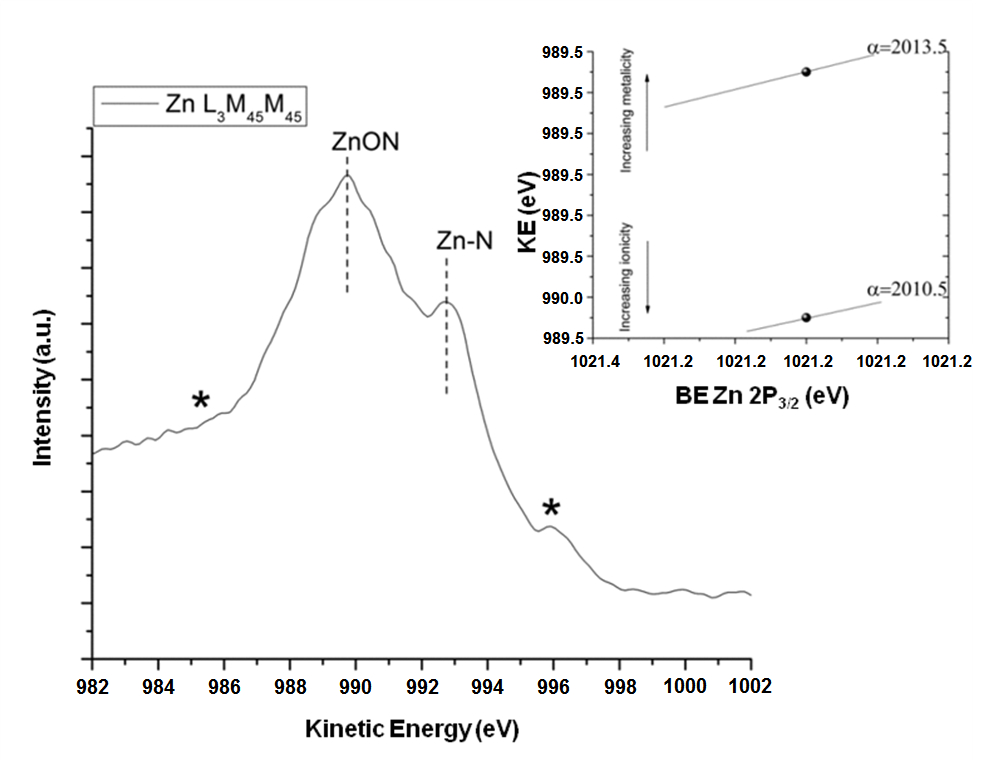


XPS Zn L_3_M_45_M_45_ Auger spectrum reveals that the film is composed of ZnN, ZnON, and ZnO species. The strong chemical effect was observed in the shape, intensity and location of the Auger lines involving deep Zn 2*p*_3/2_ (L3) and shallow Zn 3*d*_5/2-3/2_ (M45) core levels. The Auger parameters extracted from the main peak in Auger spectrum present two main straight line with the slope of -1. Each one is assigned to two distinct phases such as Zn-N in Zn_3_N_2_ and ZnON. As a first approximation the main peak of each chemical group can be symbolized by a dashed line and assigned to the ZnN, ZnON and ZnO species, respectively.

**S4. Depth profile atomic percentage of nitrogen, oxygen and zinc from XPS quantitative analysis performed after the 1^st^, 8^th^, 15^th^, 23^rd^ and 29^th^ day**


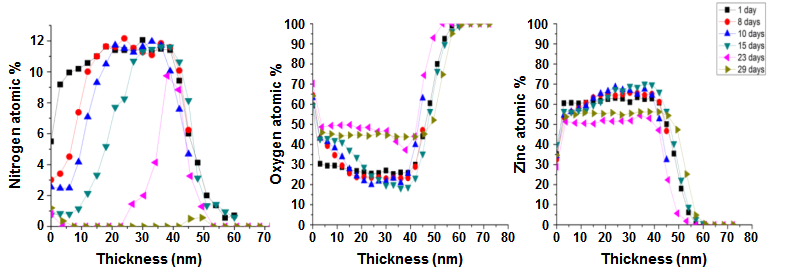


**S5. Reliability characteristics of various TFTs**

(a) ΔV_th_ of TFTs with bias illumination stress time. (b) ΔV_th_ of TFTs with light intensity.

For the bias-illumination stress measurement, we used a gate bias of 20 V and a drain bias of 10 V to each TFT device at room temperature. A white-light LED with a tunable luminance was used in order to supply visible light.
